# Clinical and hematobiochemical response in canine monocytic ehrlichiosis seropositive dogs of Punjab

**DOI:** 10.14202/vetworld.2017.255-261

**Published:** 2017-02-27

**Authors:** Manasa R. Kottadamane, Pritpal Singh Dhaliwal, Lachhman Das Singla, Baljinder Kumar Bansal, Sanjeev Kumar Uppal

**Affiliations:** 1 Department of Veterinary Medicine, College of Veterinary Sciences, Guru Angad Dev Veterinary and Animal Sciences University, Ludhiana, Punjab, India; 2 Department of Veterinary Parasitology, College of Veterinary Sciences, Guru Angad Dev Veterinary and Animal Sciences University, Ludhiana, Punjab, India

**Keywords:** dogs, *Ehrlichia canis*, ImmunoComb test, morulae

## Abstract

**Aim:**

As in India especially, the Punjab state sero-prevalence and distribution of ehrlichiosis in relation to clinico-hematobiochemical response remains largely unexplored. Thus, this study was designed to determine the prevalence of vector (tick)-borne tropical canine pancytopenia caused by *Ehrlichia canis* through enzyme labeled ImmunoComb^®^ (IC) assay in dogs from in and around Ludhiana, Punjab. Correlation of prevalence was made with various clinico-hematobiochemical parameters.

**Materials and Methods:**

Seroprevalence study was carried out using IC^®^ test kit (Biogal, Galed Labs). The study was conducted in 84 dogs presented to the Small Animal Clinics, Teaching Veterinary Clinical Complex, Guru Angad Dev Veterinary and Animal Sciences University, Ludhiana, Punjab.

**Results:**

Out of 84 suspected dogs for ehrlichiosis, based on peripheral thin blood smear examination 12 (14.28%) cases were positive for the morulae of *E. canis* and 73 (86.90%) dogs were found positive to *E. canis* antibodies through IC^®^ canine *Ehrlichia* antibody test kit, respectively. Among the different age groups 1-3 years of aged group showed highest prevalence (41.09%), followed by the 3-6 years age group (32.87%), infection levels were lower in the <1 year of age group dogs (13.69%) and more than 6 years age group dogs (12.32%). The highest prevalence was seen in Labrador retriever. This study indicates that season plays a very important role in the prevalence of ehrlichiosis. The most common findings observed were anemia, leukocytosis, neutropenia, lymphopenia, thrombocytopenia, eosinophilia followed by hyperbilirubinemia, increased levels of aspartate aminotransferase, alanine aminotransferase and alkaline phosphatase, hypoalbuminemia, hyperglobulinaemia, decrease in albumin and globulin ratio, increase in blood urea nitrogen and creatinine.

**Conclusions:**

Serological techniques like IC^®^ are more useful for detecting chronic and subclinical infections and are ideally suited to epidemiological investigations.

## Introduction

Canine ehrlichiosis a tick-borne disease (*Rhipicephalus sanguineus*, the brown dog tick) is caused by *Ehrlichia canis* obligatory intracellular small, Gram-negative, pleiomorphic obligate intracellular cocci that infect blood cells in canines which come under vector-borne diseases affecting dogs [1]. Clinical signs vary based on acute, subclinical and chronic phase. However, the disease is mainly characterized by high fever (104-105°F), anorexia, weakness, epistaxis, lymphadenopathy, and edema of dependent parts [2].

Diagnosis is mainly based on routine blood smear examination. However, more sensitive and specific molecular and serological diagnostics techniques can be used for confirmation of cases negative by microscopy. The gold standard test for detection of canine monocytic ehrlichiosis (CME) is indirect immunofluorescence antibody (IFA) test. However, this test has to be performed in selected laboratories and requires extensive equipment and trained personnel. Whereas, the enzyme-linked immunosorbent assay (ELISA) is a semiquantitive test where small quantities of antigen were used to detect the specific antibodies. Especially the commercially available dot-ELISA kits are used to detect the *E. canis* immunoglobulin-G (IgG) antibodies [3]. Among them being the ImmunoComb (IC)^®^ (Biogal, Israel) dot-ELISA has been efficient in detecting anti-*E. canis* antibodies in sera from naturally infected dogs presenting symptoms [4].

Since not much work have been done on seroprevalence study in relation to hematobiochemical changes on ehrlichiosis in Punjab, India. Therefore, this study was conducted to investigate the serology based prevalence of *E. canis* infection and its correlation with the hematobiochemical findings.

## Materials and Methods

### Ethical approval

Permission for animal experiments has been taken by Committee for the Purpose of Control and Supervision of Experiments on Animals (CPCSEA) under the Ministry of Environment and Forests, Government of India. Further, for sample collection and to conduct other procedures, application was submitted to Instituitional Animal Ethics Committee (IAEC) for a period of 12 months and got approved to carry out the research work (Regn. No.497/GO/ab/2001).

### Study area

This study was conducted at Small Animal Clinics, Teaching Veterinary Clinical Complex, Guru Angad Dev Veterinary and Animal Sciences University, Ludhiana, Punjab, India. After complete clinical examination, 84 dogs with the signs of ehrlichiosis and reduced platelet count were screened by both blood smear examination, and IC^®^ dot-ELISA kit and samples were subjected to hematobiochemical studies.

### Hematobiochemical parameters

The collected blood samples were subjected for complete hematology (hemoglobin [Hb], total leukocyte count [TLC], differential leukocyte count, and total platelet count) by ADVIA^®^ 2120 (Hematology System, Siemens Healthcare Diagnostics Inc., USA), and serum samples were used for biochemical analysis (total bilirubin, aspartate aminotransferase [AST], alanine aminotransferase [ALT], alkaline phosphatase [ALKP], total protein, albumin, blood urea nitrogen [BUN], and creatinine) by automatic biochemical analyser (Johnson & Johnson Diagnostic Kits, Mumbai, India). Results obtained from blood smear examination, hematobiochemical studies, and serological studies were compared and analyzed to get definitive diagnosis.

### Serological detection of IgG anti-E. canis antibodies by IC^®^ canine Ehrlichia antibody test kit (Biogal, Galed Labs)

Serum samples obtained from the 0^th^ day blood samples of ehrlichiosis suspected dogs used for this study. As 0^th^ day animals were naturally infected by vector transmission presented to the clinic with the signs of ehrlichiosis. Serum samples from these animals were subjected to IC^®^ canine *Ehrlichia* antibody test (Biogal Galed Lab., Israel) on the same day at room temperature (20-25°C) and tests performed based on the manufacturer’s instructions. The sensitivity of the test is 100% and specificity is 94.1%. The test does not cross-react with other blood parasite antibodies. An equivalent intensity of the color reaction in comparison with a positive reference point was used as guide to denote the level of antibodies in each sample: Intense color reactions as compared to the reference spot were considered positive for antibodies against *E. canis*. Whereas a colorless or faint gray color reaction indicates either a negative result or undetectable levels of antibodies.

Antibody titers for the different “S” levels (IC^®^ scores) were followed as per manufactures protocol. The titers are graded as S1 and S2 (1:20-1:40), S3 and S4 (1:80-1:160), S5 and S6 (1:320-1:1280) [5]. Further to see the treatment efficacy, collected blood samples after 15^th^ and 21^st^ day of post treatment and subjected to nested polymerase chain reaction and to know the hematobiochemical improvement after 2^nd^ and 3^rd^ week of post-treatment.

### Statistical analysis

The prevalence of the disease was determined with regard to months, season, age, breed and sex in the affected animals and possible hematobiochemical alterations and possible associations between the evaluated variables and positive reaction to the agents were determined. Further, to see any statistically significant differences among various hematobiochemical parameters between the positive groups and the control group were analyzed by one-way analysis of variance at 5% level of significance using SPSS software (Tukey multiple comparison test).

## Results

### Parasitological prevalence

**Figure-1:**
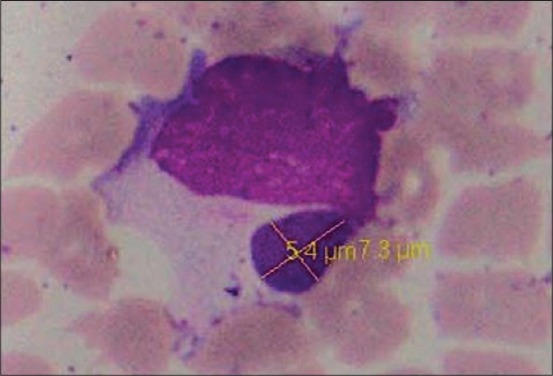
*Ehrlichia canis* morula of size 5.4 µm in monocyte of dog.

Examination of Leishman-stained peripheral thin blood smear revealed 14.28% (12/84) positivity for the morulae of *E. canis*. *E. canis* was observed as intracytoplasmic inclusion bodies of varying sizes and shapes in monocytes. The majority of morulae were homogeneous and dense inclusions and more were detected in monocytes. The most commonly encountered form was the large spherical morulae of size 5.4 µm (Figure-1).

### Seroprevalence

Among 84 suspected dogs, 73 (86.90%) dogs were seropostive to *E. canis* antibodies. High positive reaction to *E. canis* was seen in 53.57% (45/84) cases, medium positive reaction was in 22.61% (19/84) and 10.71% (9/84) cases showed low positive reaction. Negative reaction was seen in 13.09% (11/84) cases. Reactions were characterized based on the intensity of the dot developed on the comb which was cross-matched with the combscale. The titer was graded according to the “S” levels on combscale on matching. It is a dot-ELISA detected by naked eye. Results were shown in Table-1 and interpreted according to standard data provided in the instructional manual with the ELISA-kit (Table-2). All blood smear positive cases (12) were also found to be seropositive.

**Table-1:** Screening of *E. canis* suspected cases by using IC^®^ canine *Ehrlichia* antibody test kit (Biogal, Galed Labs.).

Score	Titre	Tooth number and results (n=84)	Percentage
≥S5	1:320-1280	Number of high positive reaction to *E. canis*	53.57
		45	
S3-S4	1:80-1:160	Number of medium positive reaction to *E. canis*	22.61
		19	
S1-2	1:20-1:40	Number of low positive reaction to *E. canis*	10.71
		9	
S0	Nil	Negative reaction	13.09
		11	

n=Number of dogs screened for ehrlichiosis, IC=ImmunoComb, *E. canis=Ehrlichia canis*

**Table-2:** Interpretation of IgG antibody results and titers.

Tooth No.	Results		Remarks
1	≥S5	High positive reaction to *E. canis*	Titer 1:320-1280
2	S3-S4	Medium positive reaction *E. canis*	Titer 1:80-1:160
3	≥S5	High positive reaction to *E. canis*	Titer 1:320-128
4	S-12	Low positive reaction to *E. canis*	Titer 1:20-1:40
5	S0	Negative reaction to *E. canis*	Negative
6		No positive reference*	Invalid test
7		No positive reference*	Invalid test
8	S0	Negative reaction to *E. canis*	Negative
9		High background color - interferes with reading*	Invalid test
10	≥S3	Positive reaction with high background	Positive
11	S3-S4	Medium positive reaction *E. canis*	Titer 1:80-1:160
12	S1-2	Low positive reaction to *E. canis*	Titer 1:20-1:40

IgG=Immunoglobulin-G, *E. canis=Ehrlichia canis*

### Age-wise and sex-wise prevalence

Among the different age groups, 1-3 years of age group showed highest prevalence (41.09%), followed by the 3-6 years age group (32.87%), infection levels were lower in <1 year of age (13.69%) and >6 year age group dogs (12.32%). Higher prevalence was recorded in males (71.23%) in comparison to females (28.76%).

### Breed-wise distribution

**Figure-2:**
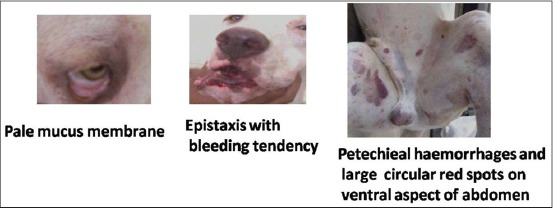
Indian bully breed of dog showing signs of ehrlichiosis.

The highest prevalence was seen in Labrador retriever. A case was showing clear signs of ehrlichiosis positive by both microscopy and serology showed high positive titer to *E. canis* antibodies (1:320-1280) (Figure-2). Usually, in study area and within the study period, Labrador retriever and German Shepherd dog (GSD) breeds were presented more to the clinics. So not much significance can be found on seasonal distribution of infected dogs in terms of breed wise. The detailed distribution and number of dog breeds seropositive to *E. canis* are shown in Table-3. Five cases of Labrador retriever, four cases of GSD were of aged between 2 and 5 years and two cases of Pomeranian 3 years of age found negative by serology.

**Table-3:** Breedwise seroprevalence of ehrlichiosis in dogs.

Breed	Seropositive cases (n=73)	Percent positive
Labrador retriever	33	45.20
GSD	11	15.07
Pug	5	6.84
Saint Bernard	5	6.84
Rottweiler	2	2.73
Dalmatian	1	1.36
Great Dane	1	1.36
Pomeranian	4	5.47
Boxer	2	2.73
Indian Bully	1	1.36
Non descript	1	1.36
Pit Bull	2	2.73
Cocker Spaniel	2	2.73
Spitz	1	1.36
Beagle	1	1.36
Bull terrier	1	1.36

GSD=German shepherd dog

### Season-wise distribution

This study indicates that season plays a very important role in the prevalence of ehrlichiosis. This study shows a significant relation between the various seasons and the prevalence of the disease. Most of the cases seen in rainy season (50.68%) followed by summer (27.39%), autumn (12.32%), and least in spring (9.58%). No cases were reported in winter, which indicates a decrease in prevalence with a decrease in ambient temperature (Table-4).

**Table-4:** Season wise seroprevalence of ehrlichiosis in dogs.

Seasons	Total number of cases (84)	Seropositive cases (73)	Seronegative cases (11)	Percent positive	Percent negative
Summer	22	20	2	27.39	18.18
Rainy	40	37	3	50.68	27.27
Autumn	12	9	3	12.32	27.27
Winter	0	0	0	0	0
Spring	10	7	3	9.58	27.27

### Clinical findings in serologically positive dogs

Dogs positive by serology were having clinical signs such as fever (38.35%), pale/congested mucus membrane (32.87%/34.24%), tick infestation (60.27%), melena (42.00%), anorexia (42.46%), lymphadenopathy (41.09%), loss of weight (36.98%), inappetance (38.35%), vomiting (24.65%), respiratory distress (15.06%), epistaxis (24.65%), petechieal hemorrhages (5.47%), depression (4.10%), bleeding tendency (6.84%), ocular discharge (6.84%), edema of legs (1.36%), and lethargy (8.21%) (Table-5).

**Table-5:** Clinical findings in serologically positive dogs.

Clinical signs/findings	Total number of samples examined (84)		Percent positive	Percent negative

	Positive by serology (73)	Negative by serology (11)		
PMM	24	2	32.87	18.18
CMM	25	5	34.24	45.45
Inappetance	28	4	38.35	36.36
Anorexia	31	2	42.46	18.18
Tick infestation	44	8	60.27	72.72
Fever	28	7	38.35	63.63
Weakness	35	5	47.94	45.45
Depression	3	0	4.10	0
Lethargy	6	0	8.21	0
Vomiting	18	0	24.65	0
Melen^a^	31	3	42.46	27.27
Respiratory distress	11	3	15.06	27.27
Lymphadenopathy	30	4	41.09	36.36
Loss of weight	27	4	36.98	36.36
epistaxis	18	3	24.65	27.27
Bleeding tendency	5	0	6.84	0
Occular discharge	5	0	6.84	0
Corneal opacity	4	0	5.47	0
Hindlimb weakness	11	2	15.06	18.18
Oedema of legs	1	0	1.36	0
Seizures	4	0	5.47	0
Petechieal hemorrhages	4	0	5.47	0

CMM=Congested mucus membrane, PMM=Pale mucus membrane

### Vital body parameters

The mean±standard deviation values of rectal temperature (°F) of seropositive dogs (104.13±1.52°F) showed significant difference from the rectal temperature of control group (92.01±0.71°F). Whereas no significant difference in heart rate and respiration rate were noted between the infected group and control group (Table-6).

**Table-6:** Study of vital body parameters in dogs positive for ehrlichiosis.

Parameters (0 day)	Rectal temperature (°F)	Heart rate (per minute)	Respiration rate (per minute)	Pulse rate (per minute)
Sero positive cases (n=73)	104.13±1.52^b^	89.17±11.01^a^	29.36±7.13^a^	87.58±10.97^a^
Control (n=10)	92.01±0.71^a^	90.3±11.69^a^	29.8±2.15^a^	88.8±11.50^a^

^a,b^: 5% level of significance

### Hematobiochemical findings

The most common findings observed were anemia, leukocytosis, neutropenia, lymphopenia, thrombocytopenia, eosinophilia followed by hyperbilirubinemia, increased level of AST, ALT and ALKP, hypoalbuminemia, hyperglobulinaemia, decrease in albumin and globulin ratio, increase in BUN and creatinine. A significant decrease in the mean values of Hb, packed cell volume (PCV), platelets, albumin and globulin ratio and increase in the mean values of AST, ALT, ALKP globulin and creatinine were found (Tables-7 and 8).

**Table-7:** Hematological findings in serologically positive dogs for ehrlichiosis.

Parameters (0 day)	Hb (g/dL)	TLC (10^3^/µL)	TEC (10^6^/µL)	PCV (%)	Platelet (10^5^/µL)	Neutrophils (%)	Lymphocytes (%)	Eosinophils (%)
Sero positive (n=73)	8.98±2.48^a^	12.73±7.05^a^	4.54±2.56^ab^	26.98±7.61^ab^	0.56±0.40^a^	77.53±15.55^a^	21.08±15.17^a^	1.36±2.06^a^
Control (n=10)	13.19±0.76^b^	8.56±1.106^a^	6.3±0.55^b^	37.94±3.710^c^	3.23±0.45^b^	72.5±2.66^a^	26.52.89^a^	2.3±1.16^a^

Figures with different superscripts in a column differ significantly at p<0.05, Hb=Hemoglobin, TLC=Total leukocyte count, TEC=Total erythrocyte count, PCV=Packed cell volume

**Table-8:** Biochemical findings in serologically positive dogs for ehrlichiosis.

Parameters (0 day)	Total bilirubin (mg/dL)	AST (U/L)	ALT (U/L)	ALKP (U/L)	TP (g/dL)	Albumin (g/dL)	Globulin (g/dL)	A:G ratio	BUN (mg/dL)	Creatinine (mg/dL)
Sero positive (n=73)	0.91±1.77^a^	28.45±14.00^b^	58.61±28.05^b^	128.11±49.76^b^	7.18±1.83^a^	2.45±0.75^ab^	4.74±1.68^b^	0.59±0.28^a^	22±10.66^a^	1.37±0.63^b^
Control (n=10)	0.38±0.16^a^	13.8±1.55^a^	27.2±9.65^a^	80.2±15.51^a^	6.1±0.61^a^	3.00±0.21^b^	3.1±0.58^a^	0.910±0.23^b^	15.5±5.21^a^	0.81±0.12^a^

Figures with same superscripts in a column do not differ significantly at p<0.05. AST=Aspartate aminotransferase, ALT=Alanine aminotransferase, ALKP=Alkaline phosphatase, TP=Total protein, BUN=Blood urea nitrogen

## Discussion

### Blood smear examination and clinical findings

Our microscopic study was agreeing with the findings of Eljadar [6] who reported 7.9% (75/951) of the cases were positive for ehrlichiosis by blood smear examination. Milanjeet [7] found 2.34% of cases to be positive for *E. canis* morulae in the same region of Punjab. Dhankar *et al*. [8] found 11.35% dogs positive for ehrlichiosis in Haryana and Delhi states. Our clinical findings in dogs with canine monocytic ehrlichiosis are agreeing with the findings of Das and Konar [9] and Sacchini *et al*. [10]. Shipov *et al*. [11] mentioned in their study about 37.5% of positive cases were having rectal temperature more than 107.25°F.

### Age- and sex-wise distribution

In this study, we have seen that 1-3 years of age group dogs showed the highest prevalence. Harrus *et al*. [12] observed disease in all age groups. Harikrishnan *et al*. [13] reported dogs aged from 15 days to 15 years were affected with ehrlichiosis indicating that all the ages of dogs are susceptible to ehrlichiosis. Abiramy *et al*. [14] observed that maximum cases of canine ehrlichiosis (36%) were observed in dogs of 5-10 years of age and maximum cases were noticed in female dogs. Costa *et al*. [15] observed male dogs more than 5 years of age had higher rates of anti-*E. canis* antibodies.

### Breed- and season-wise distribution

In this study, the disease prevalence was highest in Labrador retriever breed of dogs as compared to others (Table-1). Chandrasekar *et al*. [16] and Bhadesiya and Modi [17] also found that Labrador breed of dogs was also most susceptible. In our study, the disease was found to be most prevalent in rainy and summer followed by autumn and least in spring season. The probable reason behind this trend may be correlated to the seasonal activity of the brown dog tick, *R*. *sanguineus* was more abundant in hot and humid period of the year by Soulsby [18]. Similarly, Eljadar [6] from Ludhiana, Punjab recorded maximum prevalence of the disease during the summer season with the prevalence rate of 56% followed by rainy season (37%).

### Serological examination

Mainly our results are agreeing with the similar work previously done by Eljadar [6] in the same region of Punjab, found that 93.33% (70/75) cases were positive by serology. Harikrishnan *et al*. [13] detected *E. canis* antibodies in sera from 21 out of 56 dogs (37.5%) in ELISA and 23 dogs (41.1%) in dot-ELISA. They stated that ELISA is a valuable tool for diagnosing the subclinical and chronic forms of canine ehrlichiosis. Akhtardanesh *et al*. [19] found overall seroprevalence of ehrlichiosis was 14.63% which was determined as 13.8% and 10.6% using IFA test and rapid immunochromatography, respectively.

de Castro *et al*. [20] in their work stated that after 30 days of inoculation all the infected dogs showed positive titers for *E. canis* by testing all the samples for specific IgG response to *E. canis* with dot-blot ELISA kit (IC^®^, Biogal). Sasanelli *et al*. [21] reported a case with an antibody titer of 1:160. Castro [22] and Oria [23] used the IC test to determine IgG antibodies specific for the organism. Variable prevalence of ehrlichiosis has been reported from various parts of India. Kumar *et al*. [24] reported overall positivity for *E. canis* 6% (29/485) in canines from Chennai city. Chipde *et al*. [25] had shown 42.85% prevalence of canine ehrlichiosis in Nagpur city. Ybanez *et al*. [26] found 438/913 cases were serologically positive for *E. canis* using IC^®^ (Biogal) test kit and positive dogs produced varied clinical signs that may be influenced by the thrombocytopenic and anemic states of affected animals.

### Hematobiochemical findings of ehrlichiosis

Thrombocytopenia, anemia, hypoalbuminemia, increase in ALKP, decreased albumin and globulin ratio were the most common findings in diagnosing canine ehrlichiosis. This study depicts 100% prevalence of thrombocytopenia in *E. canis* seropositive dogs. A similar study by Bhadesiya and Modi [17] evidenced that the mean values of Hb, PCV, TEC, TLC, and total platelet count were significantly deceased in dogs which are positive by IC^®^ test kit. Sasanelli *et al*. [21] showed increased levels of ALT, AST, ALKP, BUN, creatinine and total bilirubin. Asgarali *et al*. [27] stated that thrombocytopenia is a common finding in dogs with ehrlichiosis. Akhtardanesh *et al*. [19] found 16.66% seropositive cases displayed hyperglobulinemia, thrombocytopenia, leukopenia, anemia, and high ALKP level. Kuehn and Gaunt [28] reported low albumin globulin ratio as serum biochemical abnormality in natural infection with *E. canis*. Mylonakis *et al*. [29] observed hypoalbuminemia and increased level of ALT activity in dogs with ehrlichiosis.

In summary, it can be concluded that IC^®^ canine *Ehrlichia* antibody test kit can be used for both prevalence study as well as pen side diagnostic tool in diagnosing CME, apart from the routinely used conventional methods and above-mentioned hematobiochemical alterations must be included in the differential diagnosis when these are observed during routine laboratory evaluations.

## Conclusions

Since in India, prevalence and distribution of ehrlichiosis remain largely unexplored, serological techniques like IC^®^ are more useful for detecting chronic and subclinical infections and are ideally suited to epidemiological investigations. IC^®^ canine *Ehrlichia* antibody test kit can be used as a pen-side test kit in diagnosing canine monocytic ehrlichiosis.

## Authors’ Contributions

MRK: Conducted research work and prepared manuscript: PSD: Designed research work and Procured IC^®^ antibody test kit,; LDS: Conducted microscopic examination of the blood smear, helped in preparation of manuscript; BKB: Provided research materials to carry out research work; SKU provided useful technical inputs, helped in collection of samples. All authors have read and approved the final manuscript.
